# Per‐ and polyfluoroalkyl substances thermal destruction at water resource recovery facilities: A state of the science review

**DOI:** 10.1002/wer.1483

**Published:** 2020-12-31

**Authors:** Lloyd J. Winchell, John J. Ross, Martha J. M. Wells, Xavier Fonoll, John W. Norton, Katherine Y. Bell

**Affiliations:** ^1^ Brown and Caldwell Walnut Creek California; ^2^ EnviroChem Services Cookeville Tennessee; ^3^ Great Lakes Water Authority Detroit Michigan

**Keywords:** combustion, emissions, incineration, PFAS, products of incomplete combustion, residence time, temperature, thermal by‐products, turbulence, wastewater

## Abstract

Per‐ and polyfluoroalkyl substances (PFAS) are a recalcitrant group of chemicals and can be found throughout the environment. They often collect in wastewater systems with virtually no degradation prior to environmental discharge. Some PFAS partitions to solids captured in wastewater treatment which require further processing. Of all the commonly applied solids treatment technologies, incineration offers the only possibility to completely destroy PFAS. Little is known about the fate of PFAS through incineration, in particular, for the systems employed in water resource recovery facilities (WRRF). This review covers available research on the fate of PFAS through incineration systems with a focus on sewage sludge incinerators. This research indicates that at least some PFAS destruction will occur with incineration approaches used at WRRFs. Furthermore, PFAS in flue gas, ash, or water streams used for incinerator pollution control may be undetectable. Future research involving full‐scale fate studies will provide insight on the efficacy of PFAS destruction through incineration and whether other compounds of concern are generated.

**Practitioner points:**

- Thermal processing is the only commercial approach available to destroy PFAS.
- Thermal degradation conditions required for destruction of PFAS during incineration processes are discussed.
- Fate of PFAS through water resource recovery facility incineration technologies remains unclear.
- Other thermal technologies such as smoldering combustion, pyrolysis, gasification, and hydrothermal liquefaction provide promise but are in developmental phases.

## Graphical abstract

Fate of PFAS through water resource recovery facility incinerator technologies.

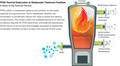

## Introduction

per‐ and polyfluoroalkyl substances (PFAS) encompass a wide range of compounds, numbering in the thousands, that have been used in a large variety of consumer and industrial products and, consequently, are widely distributed in the environment (Buck et al., 2011).

As a result of the persistence and toxicity of these compounds, the Stockholm Convention, which manages risks of persistent organic pollutants through a global legally binding instrument, has restricted production of perfluorooctanesulfonic acid (PFOS) (UNEP, 2009) and banned the production of perfluorooctanoic acid (PFOA) (UNEP, 2019). Other actions are planned for perfluorohexane sulfonyl fluoride (PFHxSF) (UNEP, 2018). The United States Environmental Protection Agency (USEPA) followed suit by releasing the PFAS Action Plan: Program Update (USEPA, 2020b), which is the first time in USEPA’s 50‐year history that it has tapped all program offices to address an emerging contaminant of concern. As a result, many of the major fluoropolymer and telomer manufacturers have committed to phasing out long‐chain polyfluorinated substances through public or private initiatives. However, international production of PFAS continues in countries such as China (Swedish Chemical Agency, 2015) that have initiated production of perfluorohexane sulfonic acid (PFHxS) precursors and other PFAS compounds as replacement products, which may present concerns of their own.

While global phase‐outs are being implemented for some of the significant long‐chain compounds, widespread distribution and health concerns (Buck et al., 2011a) have led government agencies to start regulating PFAS in drinking water. Although there are sufficient data for risk assessment of PFOA, PFOS, and several other PFAS, most PFAS detected in drinking water lack adequate data for proper risk characterization (ASTDR, 2018; Post et al., 2017). The European Union (EU) reached a provisional agreement in 2019 by setting a legally binding cumulative drinking water limit of 100 ng/L for the sum of 20 PFAS, and within 3 years regulators are mandated to develop testing protocols as well as establish a legal limit for 4,700 PFAS (The Greens/EFA in the European Parliament, 2019). In the United States (U.S.), the USEPA issued a health advisory level (HAL) of 70 ng/L for the sum of PFOA and PFOS in drinking water; however, because HALs are nonenforceable limits and USEPA’s Maximum Contaminant Level (MCL) promulgation process is expected to take years, many U.S. states are establishing their own regulatory limits for PFOA, PFOS, and others that are well below USEPA’s HAL (Cordner et al., 2019).

Research focused on understanding the sources of these compounds in drinking water has identified municipal water resource recovery facilities (WRRF) as an important pathway (Clara et al., 2008). WRRFs provide several conduits for introducing PFAS to the environment: point source discharges of effluent, leakage or unintended releases from surface impoundments or sewer systems, air emissions, disposal of biosolids, and other by‐products generated during the treatment process. PFAS are proven to exist in the effluent (Arvaniti & Stasinakis, 2015) and sludge (Eriksson et al., 2017; Hamid & Li, 2016; Lee et al., 2014) from WRRFs. Concentrations of selected PFAS increase during treatment and are generally higher in WRRF effluent than influent (Eriksson et al., 2017; Gallen et al., 2018; Kim Lazcano et al., 2019; Loganathan et al., 2007; Schultz et al., 2006; Venkatesan & Halden, 2013; Wang et al., 2018). The increases in concentration during treatment are attributed to the likelihood of precursors transforming in the wastewater treatment process (Eriksson et al., 2017; Loganathan et al., 2007). PFAS partition from wastewater and adsorb to the wastewater solids differentiated by hydrophobic and electrostatic interactions (Eriksson et al., 2017; Guo et al., 2010; Kim Lazcano et al., 2019; Loganathan et al., 2007; Nakayama et al., 2019; Pan et al., 2016). The composition of PFAS in wastewater or the solids derived thereof is a function of the WRRF treatment processes, the type and concentration of PFAS received by the WRRF, the biological and chemical transformation to intermediate and terminal degradation products, and the physical or chemical partitioning of congeners (Chen et al., 2013; Dimzon et al., 2017; Oliaei et al., 2013; Schultz et al., 2006).

Because PFAS may be concentrated in solids captured in wastewater treatment processes (Rainey, 2019), WRRFs might introduce these compounds to the environment through the land application of biosolids, potentially allowing PFAS to enter surface water through runoff or infiltrate to groundwater (Lindstrom et al., 2011). What has not been well studied is the potential for sewage sludge incinerators (SSI) to act as a source of these compounds to the environment.

In North America, there are more than 100 SSIs in operation that combust dewatered sewage sludge, or the solids generated during wastewater treatment. Thermal combustion has been reported as a critical method for destroying PFAS (USEPA, 2020c) and is important in processes such as regeneration of spent granular activated carbon (GAC) used in drinking water and in remediation treatment processes. In other words, SSIs may present a unique opportunity to destroy PFAS.

Despite the highly oxidized nature of PFOA and PFOS, these and other PFAS display a relatively high thermal reactivity (Lee et al., 2012). The temperature used for thermal incineration of PFAS in carbon regeneration is usually higher than 1,000°C (Lee et al., 2013); however, in laboratory studies, more than 99% of PFOS is degraded at 600°C (Taylor & Yamada, 2003). Studies have shown the required degradation temperature increases with increasing perfluoroalkyl chain lengths (Rayne & Forest, 2009).

While thermal combustion of PFAS has been studied for regeneration of spent activated carbon in oxygen‐poor atmospheres, limited information is available on the fate of PFAS through SSIs. SSIs are expected to destroy at least some of the PFAS in wastewater solids, given the available research. For example, Takemine et al. (2013) observed 90% mineralization of PFOA in an airstream at 700°C. Alkali addition enhanced destruction of halogenated compounds (Kamarehie et al., 2014; Takata et al., 2015; Yin et al., 2013), and laboratory‐scale incineration of lime‐conditioned sludge promotes fluorine mineralization from PFOS (Wang et al., 2013). Other chemical groups may serve as analogs to supplement PFAS‐specific research. For example, full‐scale waste incinerators and cement kilns have been reported to destroy chlorofluorocarbons at greater than 99.99% efficiency (Ueno et al., 1997; Urano et al., 2011).

The extent of PFAS thermal destruction (i.e., thermal degradation by‐product formation or complete mineralization) is also poorly understood. No published data currently exist on the overall fate of PFAS through an SSI, although limited information from other industries can be referenced. The primary point of release from an incineration system is the flue gas emitted from the stack, where any recalcitrant PFAS or by‐products from the incineration process would be released directly to the environment. Some researchers have shown that off‐gas from incineration of PFAS‐containing textiles emitted no detectable PFOA (Taylor et al., 2014). Conversely, García et al. (2007) observed significant PFAS emissions, roughly 22% of the parent compound on a carbon basis, in laboratory‐scale studies from thermal degradation of polytetrafluoroethylene (PTFE) under substoichiometric oxygen conditions at temperatures ranging from 750 to 1,050°C. Recently, air concentrations of PFAS were determined at, and upwind of, municipal solid waste (MSW) incineration plants. Higher concentrations were found at the facilities than upwind (Wang et al., 2020).

Tracking the fate of PFAS through any system is, in part, limited by available sampling and analytical methods. Agencies developing standardized methods for analyzing specific PFAS congeners include USEPA, American Society for Testing and Materials (ASTM), and the International Organization for Standardization (ISO). In the case of SSIs, these methods need to be adapted for the solids or aqueous phase samples that represent the inputs/outputs of the incineration process. Current analyses only measure a fraction of the PFAS present, partially due to the limited availability of analytical standards. Other nonquantitative or surrogate indicator approaches, such as nontargeted or total fluorine analyses, could be used to elucidate the fate of PFAS through an incineration system.

This literature review aims to identify the current understanding regarding the fate of PFAS through SSI systems. With this review, the WRRF owners will have a comprehensive understanding of the state of the art of PFAS thermal behavior and of approaches likely to be useful in understanding their fate through SSIs.

## PFAS Diversity

The following introduction to the PFAS chemical family is intended to help the reader better understand the fate of PFAS through an SSI. This introduction includes basic chemical structure, terminology, and classification within the PFAS family.

**Figure 1:**
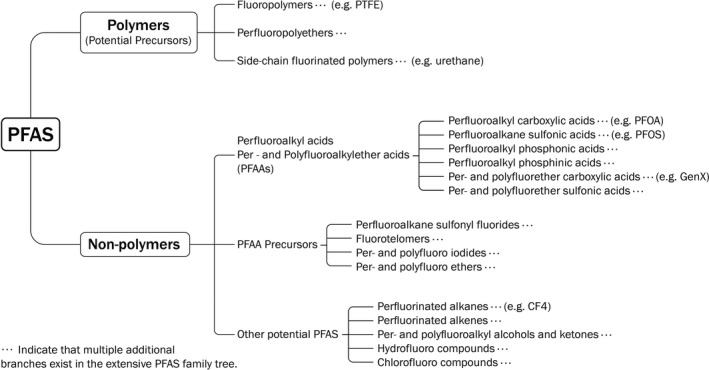
PFAS family schematic.

Every PFAS contains a common structural element, the perfluoroalkyl group (C_n_F_2n+1_) (Buck et al., 2011b; Horst et al., 2018) and has a linear or a branched alkyl chain (Kissa, 2001). PFAS comprise an extensive family tree, the roots of which are illustrated in Figure 1. The USEPA has compiled a consolidated master list of nearly 8,000 chemicals that fit into the PFAS category (USEPA, 2020d). Beyond the scope of this review, more comprehensive schematics of PFAS families of compounds are found in the literature (ITRC, 2020; OECD, 2018; Wang et al., 2017).

The primary familial classification is between the polymer and nonpolymer types of PFAS. Polymeric PFAS are potential precursors of nonpolymeric PFAS when they degrade. Nonpolymer PFAS are subdivided into perfluoroalkyl acids (PFAAs), PFAA precursors, and other potential PFAS (Figure 1). Two of the most well‐known PFAA members are PFOA, an example of the perfluoroalkyl carboxylic acid (PFCA) family, and PFOS, an example of the perfluoroalkane sulfonic acid (PFSA) family.

PFAS chemical structure and characteristics are diverse. Typical functional groups in PFAS include OH, CO_2_H, Cl, O, N, and SO_3_H. PFAS congeners exhibit many different chemical properties. PFAS can differ in polarity—polar or nonpolar; charge state—neutral, anionic, cationic, or zwitterionic; and volatility—volatile, semi‐volatile, or nonvolatile.

The alkyl carbon atoms in perfluoroalkyl substances are fully fluorinated, whereas they are not fully fluorinated in polyfluoroalkyl substances. In perfluoroalkyl substances, a hydrophilic functional group such as –CO_2_H or –SO_3_H links to the hydrophobic C_n_F_2n+1_ group; however, in polyfluoroalkyl substances, the C_n_F_2n+1_ group connects to at least one nonfluorinated alkyl carbon (–C–H) linking the perfluorinated group and the hydrophilic functional group (Dauchy, 2019; Pancras et al., 2016). Polyfluoroalkyl substances comprise a far more diverse group than the perfluoroalkyl substances (Ross et al., 2018), and polyfluoroalkyl substances can degrade into perfluoroalkyl substances (Dauchy, 2019). Another emerging subclass of PFAA compounds is the perfluoroether carboxylic acids; GenX, the ammonium salt of hexafluoropropylene oxide dimer acid (HFPO‐DA) fluoride, introduced to the commercial market as a replacement for PFOA, is an example.

Other PFAA precursors can generate PFAA compounds during processing and in the environment. One such subgroup is the fluorotelomers, which are polyfluorinated molecules with an ethyl (–CH_2_–CH_2_–) group between the fully fluorinated carbon chain and a variety of different functional groups. For example, the 8:2 fluorotelomer alcohol has a 2‐carbon ethyl alcohol group attached to 8 fluorinated carbons.

## Thermal Processes

Thermal processes utilize energy in the form of heat to transform materials. There are several types of thermal processes relevant to wastewater solids processing and which are typically identified by the involvement of oxygen in the process.

Combustion entails a chemical reaction in which an oxidant, typically oxygen, reacts with a reducing agent (fuel). The chemical reaction breaks apart chemical bonds of the reactants to form more thermodynamically stable end products. In hydrocarbon combustion, the carbon (C)/hydrogen (H) fuel component combines with oxygen to form carbon dioxide (CO_2_) and water (H_2_O), If the reaction is exothermic, the released energy often is sufficient to self‐sustain the process once the initial activation energy is provided. SSIs use combustion to process, or stabilize, wastewater solids. The term “incineration” generally refers to combustion of a waste product, so while wastewater solids are often utilized as an energy source, combustion of these solids is typically referred to as incineration.

In the absence of oxygen, or at temperatures lower than required for combustion, materials will still break down when exposed to heat. In the strict absence of oxygen in the chemical reaction, the thermal process is called thermolysis. Calcination of limestone (CaCO_3_) to quicklime (CaO) using heat to drive off CO_2_ is a simple thermolysis example. Pyrolysis and gasification, examples of thermal processes in oxygen‐limited environments, have been sparingly applied to wastewater solids. These processes are managed to generate simpler hydrocarbon substrates for subsequent use.

## Overview of Sewage Sludge Incinerators and Related Thermal Technologies

Wastewater solids are currently processed by several thermal technologies while others are in development. The following describes the dominant combustion technologies employed at SSI facilities, industry analogs, and alternative technologies emerging in the industry.

### Incinerators

Sewage sludge incinerators are a subset of incineration applications, including municipal solid waste incineration, cement kiln co‐incineration, and hazardous waste incineration. Detailed discussions can be found in several sources (Albertson, 1992; Niessen, 2002; WEF, 2009).

Municipal WRRFs typically have used two furnace technologies to combust solids captured from liquid treatment. Multiple hearth furnaces (MHF) have a long track record at WRRFs with installations first constructed in the 1930s. The first fluidized bed furnace (FBF) was installed at Lynwood, Washington, in 1965. Other furnaces types have been used, but MHF and FBF overwhelmingly represent the combustion technologies in service today at WRRFs. Table 1 summarizes the key operating characteristics of SSIs compared to other incinerators.

**Table 1:** Comparison of incinerator technology operating conditions

Incinerator type	Temperature	Residence time	Turbulence	Excess Air
MHF	Upper hearths – 300–500°C Combustion hearths – 700–1,000°C Bottom hearths – 150–300°C	Solids – approximately 1 h Gases – several seconds	Intense	50–125%
FBF	Sand bed – 700–800°C Freeboard – 800–900°C	Solids – <1 min Gases – 6–10 s	Extreme	40%
Rotary Kiln	Kiln – 650–1,300°C Afterburner – 1,000–1,300°C	Solids – 1–1.5 h Gases – several seconds	Intense	50–200%
Liquid Injection	Burner – 800–1,200°C	Gases – 0.3–2.0 s	Intense	120–250%
Moving Grate	Afterburner – 850°C	Gases – 2 s	Moderate	>200%

**Figure 2:**
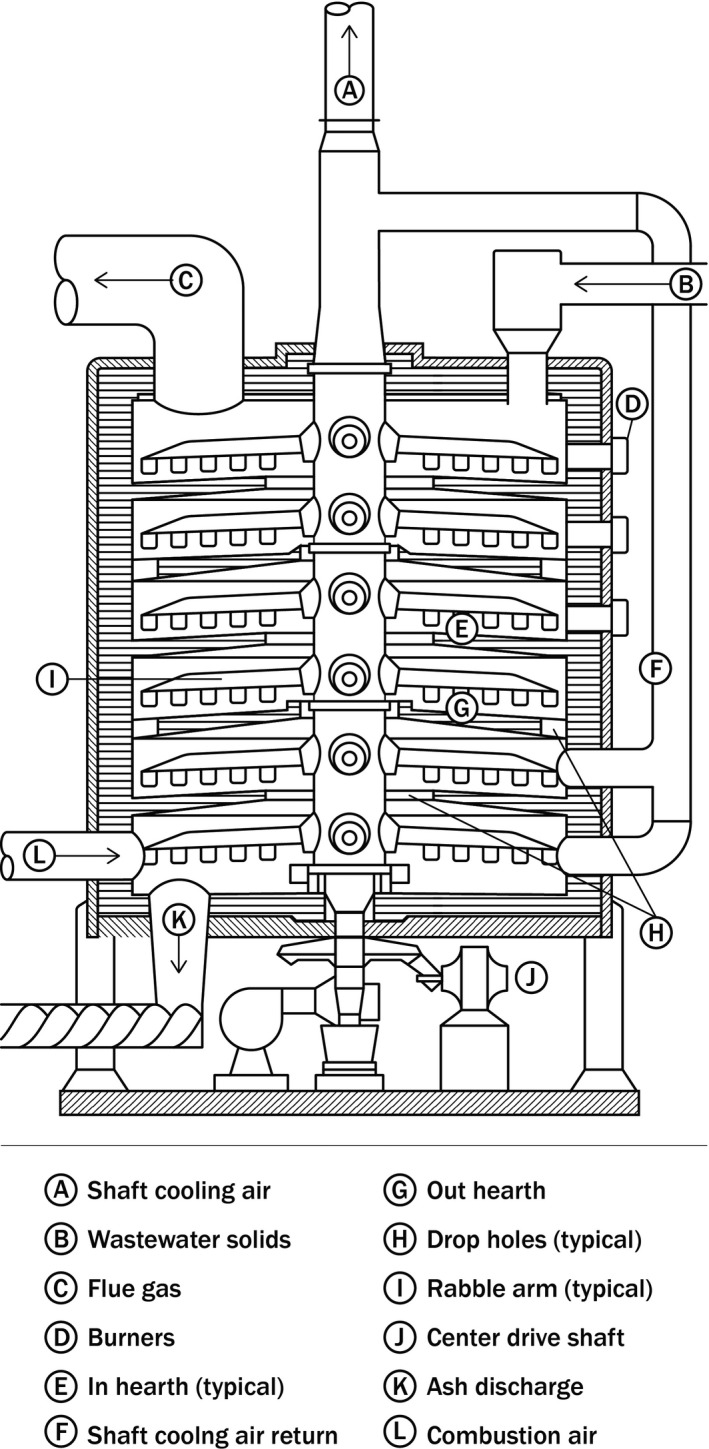
Typical MHF section view.

The MHF consists of a cylindrical steel shell arranged vertically with refractory lining and multiple levels (Figure 2). Each level, or hearth, is constructed of firebrick. A central shaft extends the full height of the furnace and supports rabble arms extending to the periphery of each hearth. The rabble arms are fixed with plows or teeth to move material inward or outward. Dewatered solids are fed to a hearth near the top of the furnace and are either moved inward or outward (movement direction alternates on each subsequent hearth) to drop through holes onto the hearth below. Water associated with the solids evaporates before the volatile fractions are released and combustion initiates. The number of hearths, which account for evaporation or combustion, vary across installations to achieve different combustion conditions. Combustion control is achieved by the speed at which the rabble arms rotate to move the solids and by burners installed on selected hearths to provide supplemental heat. Noncombustible material (i.e., ash) continues to the lower hearths, which is also where air is introduced. Combustion gases flow upward and out the exhaust ductwork on the top hearth.

The MHF provides high solids and gas retention. The combination of the gas flow and mechanical mixing of the rabble arm teeth provide an intensely mixed environment. The temperature profile of an MHF increases from the upper hearths, where water evaporates, to the middle hearths where solids combust. As ash moves to lower hearths countercurrent to the combustion air, temperatures can drop to approximately 150°C for easier handling of the ash. Combustion air requirements for acceptable performance are relatively high compared to an FBF. The higher combustion air volume, quantified as excess air which is calculated as that amount of oxygen provided in excess of stoichiometric combustion requirements, overcomes some of the comparably lower combustion efficiency in an MHF (from less turbulence) than an FBF. Most currently operating MHFs were modified after implementation of the Clean Water Act solids management rules in 1993, which lowered allowable emissions of products of incomplete combustion (PIC). Afterburners were retrofitted either externally, or by using one or more of the top hearths enabled by switching the dewatered solids introduction location to lower hearths. The afterburners typically use natural gas to elevate the flue gas temperature to achieve complete combustion. The afterburners achieve temperatures of 820°C or higher for 1–2 s.

**Figure 3:**
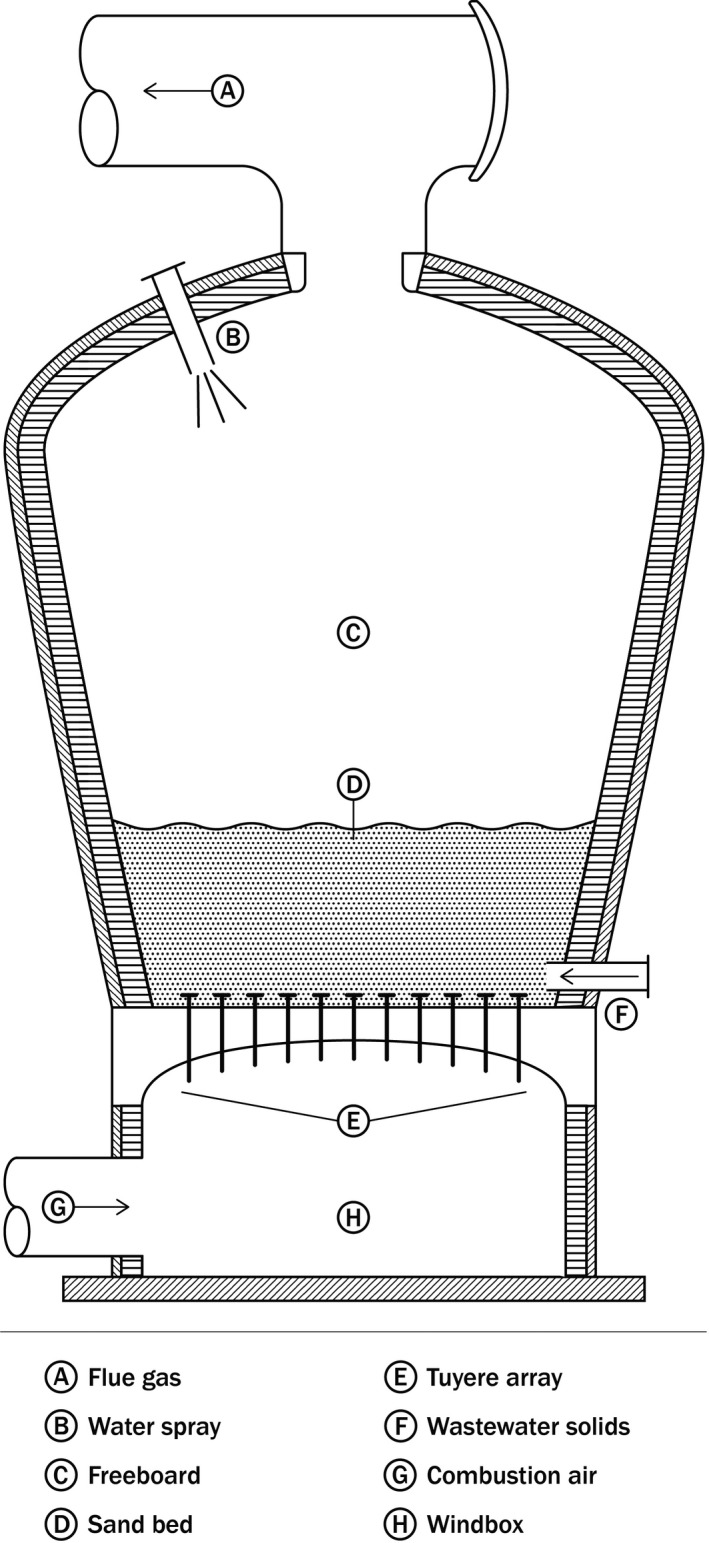
Typical FBF section view.

Fluidized bed furnaces differ significantly from MHFs in configuration (Figure 3). The FBF consists of a vertically oriented cylinder that expands in diameter with increasing height. Three major zones exist in an FBF starting at the bottom with the windbox, which acts as a plenum for the combustion air blown into the furnace. The windbox is capped by a distribution structure typically consisting of a ceiling penetrated by many tubes or tuyeres that extend through the ceiling. Head loss through the tuyeres provides uniform airflow distribution into the second zone, or sand bed, that uses the windbox ceiling as a floor. The flow of air through the three to six feet of sand creates enough drag force to fluidize the material, creating an extraordinarily turbulent or violent environment. Dewatered solids are injected into or immediately above the sand bed. Intense mixing of the sand bed facilitates heat transfer and fuel/air interaction to provide uniform temperatures and thus achieve efficient combustion. FBFs have two significant advantages over MHFs—the comparably lower excess air required, and the ability to operate without supplemental fuel, or autogenously, where dewatered solids provide all the heat needed to maintain temperatures. To operate autogenously, an FBF typically must be coupled with a heat exchanger that preheats combustion air using the waste heat from the furnace flue gas.

The third zone in an FBF is the freeboard, which extends from the top of the sand bed to the furnace roof where an outlet duct exhausts the flue gas. Volatile compounds in the dewatered solids or fixed combustible material ejected from the sand bed combust in the freeboard, resulting in a higher temperature than in the sand bed. A well‐operated FBF will maintain the freeboard temperature no more than 100°C higher than the sand bed. The expanding shell of the FBF, most notably in the freeboard region, achieves high gas residence times. To protect downstream equipment, the FBF roof is equipped with spray water to drop the exhaust temperature to roughly 850°C.

FBFs achieve lower emissions than MHFs because of the highly turbulent sand bed and uniform temperatures that minimize localized cold spots that can result in incomplete combustion. The most recent federal regulations (Standards of Performance for New Stationary Sources & Emission Guidelines for Existing Sources, 2011) provide more stringent emission limits for FBFs compared with MHFs. From this, one can infer the performance differences from FBFs compared to MHFs.

The dynamics in the FBF and MHF differ significantly from furnaces typically employed to treat hazardous or municipal solid wastes. Rotary kiln furnaces handle solid materials well and often are paired with an afterburner to combust volatile compounds. Operating conditions can be analogous to an MHF (Oppelt, 1987). Municipal solid waste can also be incinerated in a sloped moving grate‐type furnace (USEPA, 2020a). The EU mandates temperatures shall reach 850°C for 2 s (United Kingdom Department for Environment Food & Rural Affairs, 2013). Niessen (2002) provides an extensive discussion on grate type furnaces. Liquid injection hazardous waste furnaces spray or atomize particle‐free waste into a burner flame or mix the waste with the supplemental fuel before the burner with operating conditions typical of those used in afterburners coupled with other incineration technologies (Oppelt, 1987). Neither the rotary kiln nor moving grate furnaces will match the turbulence of an FBF and may be more tranquil than an MHF. Long residence times and turbulent conditions in FBFs and MHFs favor more complete combustion. As a result, the combustion efficiency may be higher in SSIs for a given temperature compared with hazardous or municipal solid waste incinerators.

Current incineration systems are required to meet emissions criteria with extensive air pollution control measures. All SSIs in the United States known to the authors employ some type of wet scrubber for air pollution control. Sandblom (2014) hypothesized that any PFAS compounds escaping the furnace would be captured in the wet scrubber due to their low pK_a_ values. Many SSIs also include equipment to remove mercury from the flue gas, which requires an activated carbon or sorbent polymer composite (SPC) system. Activated carbon removal of PFAS is well accepted for drinking water applications, and removal can be expected from the gas phase, as well. The SPC mercury removal system has yet to be investigated for PFAS removal efficiency. Some facilities use wet electrostatic precipitators downstream of the wet scrubber to capture fine particulates that could include adsorbed PFAS compounds. Currently, no published work on PFAS removal within an incineration application across the stated pollution control equipment exists.

### Emerging thermal treatment techniques

Several alternative thermal treatment processes for wastewater solids are currently in development or being implemented at a limited scale. Drivers for the evolution of these technologies have been the improved economics of wastewater solids management and energy efficiency; however, they are currently gaining increased attention for their potential as alternative PFAS destruction technologies. However, to date, none of these techniques have been proven to be commercially viable for widespread application with wastewater solids.

### Thermal drying with combustion systems

Wastewater solids can be dried to reduce mass and create a beneficial reuse product. When dried, wastewater solids have a substantial heating value (14,000–21,000 kilojoules per kilogram), similar to that of low‐grade coal (Heidrich et al., 2011; NACWA, 2010). Dried product furnaces have been installed in drying facilities to combust the dried product and capture the resulting heat for recycling back to the drying process. These systems contain a primary combustion chamber where dried product is combusted at temperatures of 760–980°C, and typically postcombustion of the flue gas (at temperatures up to 1,150°C) is performed before treatment and exhaust. The units currently are regulated under the same air emissions requirements as SSIs and require substantial air pollution control processes, typically including urea, alkaline, coke dosing, and textile filtration. Viswanathan et al. (2020) reported on progress in development of a laboratory‐scale combustion reactor designed to simulate oxidative conditions similar to those occurring in a commercial dried product furnace to measure PFAS destruction, with publication of the results expected shortly.

### Pyrolysis and gasification

Pyrolysis and gasification are thermal decomposition processes that convert solid carbon feedstocks into a combustible gas. Pyrolysis submits carbonaceous materials to high temperatures (200–1,100°C) in the absence of oxygen and generates a carbon‐rich, porous product called biochar. Gasification introduces a limited quantity of oxidant (typically air) at high temperatures (800–1,650°C) to refine the volatile organic fraction through partial oxidation and reforming reactions while converting the solid mass to ash particles. Thermal treatment of wastes in a reductive environment has garnered interest from the waste management sector for its potential to guard against the formation of harmful oxidation by‐products such as dioxins and furans (Maric et al., 2020; Rey et al., 2016).

The basic pathway for thermal PFAS destruction in a reductive environment is hydrodefluorination (HDF). HDF is the conversion of a carbon‐fluorine (C–F) bond into a carbon‐hydrogen (C–H) bond and can be performed with a variety of reagents (and catalysts). For the reaction to proceed, the resulting element‐fluorine bond formation must be sufficiently exothermic to generate the thermodynamic compensation required for the C–F cleavage, with common reagent elements including H, silicon, or boron (Kuehnel et al., 2013). The process requires a H source for the C–H bond, which often also serves as the fluorine acceptor (Kennedy et al., 1997; Kuehnel et al., 2013). H can be produced during pyrolysis through the steam reforming reaction, where the steam released from the moisture in feed materials generate H through reactions with primary pyrolysis decomposition products (Conesa & Font, 2001; Pinder, 2012; Rey et al., 2016).

A variant on the pyrolysis process, called gas‐phase reduction (GPR), introduces hydrogen gas directly into the thermal reactor and has been used to break down chlorinated hydrocarbons like polychlorinated biphenyls (PCB) (NRC, 1996). Laboratory‐scale studies conducted in Canada to assess the effectiveness of applying GPR to dried biosolids successfully produced hydrogen‐enriched methane gas but consumed a similar amount of H_2_ during the process, indicating that many of the energy recovery benefits of pyrolysis would be offset in GPR unless a well‐functioning H recovery system was present (Pinder, 2012). Catalytic hydrogenation, reduction with metals or low‐valent metal compounds, has also been proposed for hydrodefluorination of PFAS compounds (Alonso et al., 2002; Lee & Choi, 2002). Wang et al. (2015a) demonstrated transformation efficiencies upward of 80% when submitting a mixture of PFAS and calcium compounds to thermal treatment at temperatures at 600°C and higher.

Central to the potential for PFAS decomposition in pyrolysis and gasification systems is their ability to maintain PFAS within the hot zone of the reactor before volatilizing and exiting. Organic materials introduced into a pyrolysis reactor undergo various stages of thermal decomposition due to changing feedstock characteristics and moisture content. Studies show that pyrolysis causes PFAS volatilization at temperatures below 450°C, while the volatilization of organic feedstocks can require internal temperatures of up to 600°C (Gao et al., 2020; Wang, Cousins, et al., 2015). Consequently, typical design parameters related to the residence time of organic feedstocks may not correlate to those required for PFAS destruction, and further study is required to investigate the potential for PFAS transformation concerning reactor design.

Limited experimental data are available on PFAS removal during pyrolysis and gasification. Kim et al. (2015) conducted laboratory pyrolysis experiments with wastewater solids at 300 and 700°C and found no significant change of residual PFAS concentration in the biochar; however, no discussion was provided on why PFAS were still detected, given general agreement in the literature that PFAS compounds volatilize at temperatures <700°C. A pyrolysis technology supplier recently published biochar sampling data from a system operating at 850°C that demonstrated PFAS removal to nondetect levels, indicating transformation or volatilization of the compounds (Bioforcetech, 2020). If the PFAS, or partial decomposition products, do volatilize and exit the reactor in the pyrolysis gas, they would likely be submitted to gas combustion applications for energy recovery resulting in further destruction or transformation.

While syngas produced from simpler feedstocks such as woody waste and algal biomass has been refined to produce high‐value gas or liquid fuels, wastewater solids generate a high load of inorganic contaminants that make internal combustion applications difficult. Consequently, demonstration and commercial scale systems have used a thermal oxidizer to combust the syngas and capture the heat for use in upstream drying processes or conversion to electricity through the organic Rankine cycle (ORC) process. Thermal oxidizers are typically operated to achieve temperatures of 850°C or greater for more than 2 s and can achieve a greater degree of efficiency than incineration, given their ability to introduce process gases through or very near the ignition zone. Thermal oxidizers are often permitted for 99.99% emission reduction, and a recent test report of a thermal oxidizer used to control PFAS process stream emissions from an industrial facility demonstrated compliance with this requirement (Focus Environmental Inc., 2020). Consequently, the critical step for achieving PFAS control in pyrolysis and gasification systems may be the operation of the downstream thermal oxidizer.

### Hydrothermal liquefaction

Hydrothermal liquefaction (HTL) subjects solids to high temperatures and pressures (250–350°C, 10–25 mega Pascals [MPa]) to generate a liquid biocrude oil. The process is fed dewatered, slurried solids to generate biocrude, suitable for further refining into jet, diesel, and heavy fuels, while retaining an effluent stream with a high ammonia and chemical oxygen demand (COD) load that must be managed (Toor et al., 2011). During laboratory‐scale HTL experiments with wastewater solids, Yu et al. (2020) demonstrated varying levels of PFAS degradation based on analysis of the functional groups of the compounds and operational parameters. Greater than 99% transformation efficiency was observed for PFAS compounds with carboxylate functional groups, specifically PFOA, fluorotelomer carboxylic acid (FTCA), and fluorotelomer unsaturated carboxylic acids (FTUCA). Conversely, less than 34% degradation was observed with the sulfonic acid compound PFOS, even when the temperature was elevated to 350°C for 90 min. Both the original PFAS compounds and defluorinated intermediary products were found in the biocrude oil fraction, which presents a research need for understanding its resulting fate. The authors proposed potential application of reactive amendments to the HTL process to further promote destruction of recalcitrant PFAS compounds such as PFOS during HTL. Zhang et al. (2020) found that amending a plant biomass‐fed HTL process with potassium hydroxide (KOH) increased removal of perfluorosulfonic acids from <20% to 86%, indicating the potential efficacy of this strategy.

### Smoldering combustion

Smoldering combustion propagates thermal oxidation by the diffusion of an oxidant (air) through the surface of a condensed liquid or solid fuel. The process can be engineered by mixing fuel with an inert media like sand to promote mass and thermal transfer and introduce a forced, upward airflow (Wang, 2017). Rashwan et al. (2016) reported that self‐propagating smoldering combustion can be achieved with wastewater solids having a solids content as low as 20% by modulating the forced airflow to account for process and feed characteristic fluctuations. Major (2019) investigated smoldering combustion of PFAS‐laden activated carbon and a simulated waste soil mixture in a Department of Defense (DoD) Strategic Environmental Research and Development Program (SERDP) study. Results reported all PFAS compounds as nondetect levels from the treated product. The process achieved temperatures over 1,000°C for one to ten minutes based on separate tests and depending on the location in reactor column. Oxygen concentrations averaged 6%. Where emissions sampling was conducted for the simulated waste soil experiments, 82% of the available fluorine was captured as hydrogen fluoride (HF). The authors acknowledged the remaining available fluorine fraction indicated incomplete PFAS destruction. The presence of both parent PFAS compounds and fluorinated by‐products with one less functional group was identified by emissions sampling. However, compounds over nine carbons were attributed to material impurities and not by‐products. A more recent study was conducted using municipal biosolids and demonstrated PFAS removal to nondetect levels in the solid by‐product, although no gas‐phase analysis was performed (Kinsman et al., 2020).

## Documented PFAS Thermal Behavior

A significant body of literature exists on PFAS thermal behavior but focuses mostly on laboratory‐scale experiments and only considers specific PFAS congeners given analytical limitations. The following organizes the available literature considering first the early theoretical work. Subsequently, this review presents laboratory work used as a basis for current incineration guidelines, full‐scale incineration studies, and finally a summary of by‐products observed during thermal processing.

### Theoretical combustion requirements

For a combustion process to achieve complete PFAS thermal destruction (mineralization), PFAS compounds would have to be driven to their thermodynamic endpoints of CO_2_, H_2_O, HF, or sulfur compounds, if present. The introduction of additional chemical compounds such as salts, minerals, and halogens result in other end products, for example, HF as an end‐product from organic fluoride compounds. Additionally, some inert fraction, or ash, of the fuel, or waste, remains as a solid product.

The combustion process, while seemingly simple, involves thousands of elementary physical and chemical reactions, reaction kinetics, fluid dynamics, and heat transfer mechanisms (Burgess et al., 1995; Reed, 1978). Residence time, turbulence (mixing), and stoichiometry (the relative mixture of waste to fuel, oxygen, and other gas‐phase constituents) within the flame zone all impact the completeness of the combustion process, and consequently, the temperature and destruction efficiency achieved (Lewis, 2008; Niessen, 2002).

Given the dynamic nature of the incineration process as well as varying characteristics of input waste streams, operational parameters vary over time throughout the reactor (Lewis, 2008; Tsang et al., 1998). Additionally, the potential exists for flame zone failure modes, with the most notable being thermal quenching by pockets of cold unreacted material and inadequate mixing. Consequently, theoretical investigations into thermal destruction are often based on conservative operational assumptions accounting for the occurrence of nonideal conditions in operating systems (Tsang et al., 1998). These conditions lead to the formation of thermal degradation by‐products, which are a primary concern in incineration processes.

Early theoretical investigations into the potential for thermal destruction of fluorinated organic compounds focused on one‐ and two‐carbon molecules, given the interest in their use as fire extinguishing agents (Burgess et al., 1995; Tsang et al., 1998). In these studies, thermochemical and kinetic data were compiled to develop a framework for future combustion simulation experiments. The thermal and chemical stability data show notably high bond strengths for both C–F and H–F bonds (relevant bond energies identified by Tsang et al. (1998) are provided in Table 2). A high degree of energy is required for cleavage of the C–F bond suggesting that stable fluorinated intermediary products may be produced.

**Table 2:** Relevant bond energies adapted from Tsang et al. (1998)

Bond	Energy, kJ/mol	Bond	Energy, kJ/mol
CF_3_─F	552	H─H	436
CF_2_─F	352	OH─H	499
CF─F	508	CH_3_─H	439
H─F	569	CCl_3_─Cl	288
F─F	159	H─Cl	431
HO─F	216	HO─Cl	235
O─F	220	Cl─Cl	242
CF_3_─H	456	CCl_3_─H	392
CF_3_─CF_3_	408	CCl_3_─CCl_3_	301
CH_3_─H	439		

The relative stability of intermediary compounds results in less favorable reaction kinetics, which can interrupt or terminate flame chain propagating steps, resulting in the flame‐retardant properties of PFAS. Additional research postulated that, due to the relative stability of the compounds, initial decomposition requires unimolecular bond cleavage because fluorinated organics would be less susceptible to bimolecular attack (Tsang et al., 1998). As the fluorinated organics become less saturated, they are more vulnerable to radical attack and reforming reactions. However, Tsang et al. (1998) showed fluorinated organics followed the rule of hydrocarbons in a homologous series—as larger compounds break down into smaller components, those compounds increase in stability. They calculated theoretical temperatures required to achieve 99.99% destruction of various intermediary products in 1 s via unimolecular decomposition, and perfluoromethane (CF_4_) resulted in the highest predicted temperature required at 1,441°C. Destruction of CF_4_ has been noted in other studies to require temperatures ranging from 1,200 to 1,400°C (Beu, 2005; USEPA, 2020c). Given this finding, CF_4_ has been proposed as a potential surrogate for monitoring emissions from fluorinated organics incineration. Although using CF_4_ alone may underpredict PFAS destruction efficiency, multiple surrogate compounds should be considered, with representatives covering the diverse chemical properties (i.e. volatility, polarity, and ionic charge) of the chemical family.

### Thermal destruction guidelines and experimental basis

Most published research and industry guidance states that complete destruction of PFAS requires operation at the higher temperature ranges (>1,000°C), as summarized in Table 3. Notably, this guidance is based on limited conceptual or laboratory‐scale experiments and precedence on previous guidance established for hazardous waste incineration.

**Table 3:** Recent guidance and literature basis for PFAS thermal destruction

Source	Temperature noted	Commentary
Pancras et al. (2016)	1,000–1,200°C	High‐temperature incineration is required for complete PFOS degradation
Kucharzyk et al. (2017)	1,000°C or greater	High‐temperature incineration is required to destroy PFAS adsorbed to spent activated carbon
USEPA (2020c)	1,000°C	Studies found PFOA is removed to nondetect levels using laboratory‐scale combustion experiments
UNEP (2019a)	1,100°C	Combustion at hazardous waste incineration process parameters (2 s residence time at temperature) is the most appropriate way to handle PFOS waste
Ross et al. (2018)	1,100°C	High temperatures are required for destruction of gas‐phase PFAS
ITRC (2020)	1,000°C or greater	PFAS destruction can be achieved at high temperature

The baseline research for these recommendations stems from USEPA and other international environmental agency activities. Under the Toxic Substances Control Act (TSCA), the USEPA worked to identify and reduce PFAS exposure from industry, including working with 3M to phase out the use of PFOS in products and facility emissions beginning in 2001 and continuing with the 2010/2015 PFOA Stewardship Program. After 3M’s announcement to phase out the manufacture and use of PFOS, the U.S. and the United Kingdom led efforts within the Organization for Economic Co‐operation and Development (OECD) to perform a hazard assessment of PFOS in cooperation with other member countries and industry. A final draft of the assessment was published in 2002 and noted that laboratory combustion studies were being conducted on PFOS and two polymeric formulations to assess combustion by‐products over a range of temperatures (OECD, 2002). A final report was published the following year (Taylor & Yamada, 2003) discussing results of a simulated hazardous waste incineration experiment with limited air to account for nonideal combustion conditions. The chemicals were submitted in gaseous form to secondary combustion zone temperatures of 600 and 900°C using methane as the fuel source (primary reactor temperatures of 1,250°C were used to ensure volatilization). Experiments showed <0.4% and 0.05% of the PFOS fed to the reactor were detected in the exhaust at tests conducted at 600 and 900°C, respectively, indicating a high degree of removal. While a variety of small molecular weight PICs were identified at 600°C, the detection of perfluorinated alkanes was limited to C_1_ and C_2_ compounds. Two eight‐carbon perfluoroalkyl sulfonamides were also studied to see if they acted as precursors for PFOS after combustion. No PFOS was detected, but some tests detected PICs identified as benzene, tetrafluorosilane, and difluorodimethylsilane. Taylor and Yamada (2003) did not expect reformation of PFOS or other long‐chain PFAS because the methane fuel provided hydrogen atoms to scavenge fluoride radicals. Yamada et al. (2005) later published a similar laboratory‐scale study that considered the combustion of PFAS‐impregnated textiles at 1,000°C, 85% excess air, and a 2 s residence time. The focus of the study was to ascertain whether combustion of the PFAS‐impregnated material would result in PFOA emissions. Neither PFOA nor PICs were detected under nonideal combustion performance measured by a high carbon monoxide emission of 650 part per million (ppm). HF was not detected, but silicon tetrafluoride (SiF_4_) was, suggesting the HF was reacting with the silica‐based lining of the reactor.

Consideration has also been given to incineration of activated carbon used for removing fluorinated alkyl compounds from water. A review conducted by Schultz et al. (2003) reported industry correspondence stating that incineration of the saturated activated carbon for 2 s with a combustion chamber reaching temperatures of 1,200°C was sufficient for destruction, and the exhaust gas could be scrubbed to produce a solid CaF_2_ precipitate. Taylor et al. (2014) examined whether combustion of gasified fluorotelomer‐based polymer would emit PFOA at 1,000°C, in an oxidizing atmosphere, with a 2 s residence time to reflect municipal and medical waste incinerator conditions. No detectable quantities of PFOA were measured in the exhaust gas, and qualitative HF emission suggested complete mineralization occurred.

### Full‐scale incineration studies

A few full‐scale studies have been published on the fate of PFAS compounds through incineration systems with only two considering an SSI. Loganathan et al. (2007) investigated eight PFAS compounds through two WRRFs. One of the facilities employed incineration, and while not stated in the published information, the furnace was most likely an MHF based on the facility geography. The PFAS compounds were measured in dewatered solids fed to the incinerator and in the ash. No mention was made on the operating conditions of the furnace or whether the bottom or fly ash was sampled. Findings indicated a significant removal of the measured PFAS compounds; however, some compounds were still detected in the ash in the range of single to double digit nanogram per gram concentrations. Concentrations of PFOS, PFOA, perfluorooctane sulfonamide (PFOSA), and perfluorodecanoic acid (PFDA) were all detected in the ash above method detection limits and ranged from 26% to 97% less than values measured in dewatered solids on a concentration basis, except in two samples. Interestingly, two of the ash samples yielded higher PFAS concentrations, one PFOS and one PFOSA, than in the dewatered solids. The authors did not speculate on the reasons for the increased concentrations for these two analytes.

A second study is currently underway at an SSI facility employing an FBF (MacGregor, 2020). The temperature and gas residence time within the FBF were reported at 830°C and 8 s, respectively. Samples were taken at all inputs and outputs of the SSI system and analyzed quantitatively for 28 PFAS. Only partial results were available at the time of this literature review, restricted to the solids and liquids streams around the SSI. Preliminary results show that mass flows were reduced through the SSI processes for all quantitated PFAS, with the exception of 6:2 fluorotelomer sulfonate. The degree of destruction was not characterized because water used in the air pollution control systems contained PFAS and may mask the levels emitted by the furnace. Release of the ambient air and stack emissions data in the near future will help to further determine the fate of PFAS through the SSI. The authors also noted that the inorganic fluoride content of the wet scrubber discharge water was over 10,000 times that of the influent flow plus the measured PFAS fed to the FBF, assuming mineralization. The authors suggest there are significant loads of nonmeasured PFAS being combusted through the SSI.

No other studies have been published on SSIs, but limited information can be found in other industries. Two studies investigated the behavior of polymerized PFAS through incinerators. Lemieux et al. (2007) reported on the USEPA’s study of feeding carpet treated with fluorotelomer products into a pilot‐scale rotary kiln furnace operating at 952–998°C. The emitted PFAS detected, primarily PFOA and perfluorohexanoic acid (PFHxA), did not change between operating with the carpet feed or on natural gas alone, suggesting contamination with fluoropolymers used in sampling or analytical equipment. Aleksandrov et al. (2019) investigated emissions from a pilot‐scale rotary kiln incinerator with a waste heat boiler and flue gas cleaning compliant with German emissions regulations. The incinerator was fed a mixture of PTFE and wood chips with supplemental natural gas. Two combustion conditions were evaluated with conditions in the afterburner ranging from 870°C with 4 s of residence time to 1,020°C and 2.7 s of residence time. In either condition, the combined rotary kiln and afterburner excess air was 143%. A total of 31 PFAS compounds were quantified, and 11 were detected in the air emissions but not at significantly different levels than that measured in control runs without PTFE, which suggests sampling and analytical contamination. The authors concluded that incineration of PTFE at the conditions studied would not release PFAS to the environment at measurable levels.

Japan’s Ministry of Environment (2013) released a report on PFOS behavior through a full‐scale municipal solid waste incinerator operating at 1,100°C in the rotary kiln and 900°C in the afterburner with a combined gas residence time of 8 s and solids residence time in the rotary kiln of 1.0–1.5 h. Flue gas traveled through a wet scrubber and wet electrostatic precipitator to achieve emissions standards. Canisters of firefighting foam with known quantities of PFOS were fed to the incinerator. The overall PFOS destruction efficiency was over 99.999%, considering levels emitted in the flue gas, ash residues, or scrubber water discharge. The report also mentioned that flue gas emissions of fluoric carbons from the furnace, scrubber exhaust, or stack were undetectable, but did not define which compounds were analyzed.

Sandblom (2014) sampled the various streams into and out of four municipal solid waste incinerators in Sweden operating at temperatures over 850°C. Several of the nine PFAS compounds targeted were measured in the slag and fly ash streams in the single nanogram per gram (ng/g) range with perfluorononanoic acid (PFNA) consistently dominating. Levels observed in the wet scrubber discharge ranged from nondetect to single‐digit ng/L concentrations. Stack emissions were not characterized.

More recently, PFAS destruction of greater than 99.999% for five PFAS through a thermal oxidizer and four‐stage scrubbing system was reported at a Chemours chemical facility in North Carolina (Focus Environmental Inc., 2020). The thermal oxidizer treated waste gas streams laden with PFAS at temperatures exceeding 1,000°C, though neither residence time nor PICs were noted.

A recent study investigating the incineration of PFAS contaminated soils observed extensive destruction (NRC Alaska LLC, 2019). Contaminated soils from a military installation in Alaska were incinerated through a rotary kiln fitted with a secondary combustion chamber. The kiln was operated at 425–815°C with the exact temperature depending on the soil characteristics. The secondary combustion chamber temperature ranged from 980 to 1,200°C. No mention of retention times in either the kiln or secondary chamber was noted. Exhaust gases were quenched and filtered in a baghouse. One of the two trials included a packed bed scrubber after the baghouse. Samples of the contaminated and treated soil, exhaust gases, and flue gas scrubbing water were analyzed for specific PFAS compounds. For two test trials, the PFAS was typically nondetect in treated soil samples; however, a few samples did exhibit detectable levels of PFOS or PFHxS. Emissions from the incineration systems exhibited detectable levels of various PFAS but further evaluation found the XAD traps used in the sampling train and the supply water for the scrubbing system both contained background levels of PFAS. The authors concluded the emissions results were impacted and could not completely predict the background contamination in the reported results. Given the limited data provided in the report, a destruction efficiency could not be calculated. Interestingly, inclusion of the packed bed scrubber during the second test did not change PFAS emissions suggesting the PFAS evaluated are not captured in this type of emission control equipment.

Solo‐Gabriele et al. (2020) studied PFAS in landfill leachate; three leachates came from landfills dominated by MSW ash. A statistically significant correlation between the concentration of PFAS in the leachate from landfills with ash and the operating temperature range of the incinerator (*R* ^2^ = 0.92, *p* = 0.008) was observed. The lowest total PFAS concentrations (<3,400 ng/L) were found in leachate from ash where the incinerators operated between 930 and 980°C. The incinerator with the lowest operating temperatures (760–870°C) exhibited the highest PFAS leachate concentrations (12,300–13,500 ng/L). The third landfill, dominated by MSW ash supplied by an incinerator operating in the range of 815–870°C, had total PFAS from 8,400 to 8,700 ng/L. The main difference between the two leachates from ash originating from incinerators operating in lower temperature ranges was perfluorobutane sulfonate (PFBS). The four‐carbon PFAS was highest (roughly 5,000 ng/L) in the low‐temperature versus the mid‐temperature incinerator by approximately 350 ng/L. The authors proposed the lower temperature incineration may be producing shorter chain PFAS instead of proportionately destroying all PFAS. The study did not disclose the type of furnace used at these facilities or additional operating conditions beyond temperature.

Where full‐scale incineration of PFAS‐laden wastes at hazardous waste conditions has been investigated, it is important to note that wastes are highly concentrated (e.g., spent activated carbon used for aqueous PFAS removal and aqueous firefighting foam). The Resource Conservation and Recovery Act (RCRA) labels a waste “hazardous” when it contains at least 0.1% percent of a halogenated organic chemical. For comparison, the highest levels of a PFAS compound (PFOS) identified in wastewater solids, given substantial contributions from an industrial discharger, is 0.0005% percent (Sun et al., 2011; USEPA, 2005; Yu et al., 2009).

### Thermal by‐product formation

Reports of PFAS treatment using thermal technologies on a variety of matrices provides some indication of potential thermal degradation by‐products. Many bench and pilot studies have looked at the decomposition of specific PFAS congeners and by‐product identification. By‐product identification is complicated by the broad characteristics involving polar or nonpolar, anionic, cationic, or zwitterionic, and volatile, semi‐volatile, or nonvolatile forms PFAS can take, so not all by‐products can be detected. Several studies identified thermal PFAS degradation by‐products as summarized in Table 4. These studies show that PFAS will decompose at temperatures relevant to operating conditions of SSIs although by‐product formation will be a concern.

**Table 4:** PFAS thermal degradation by‐products reported

Reference	Parent PFAS	Treatment temperature	Atmosphere	Fluorinated by‐products noted^A^
NRC Alaska (2019)	PFOS and PFOA (measured in contaminated soil sample)	425–815°C (kiln) 980–1,200°C (secondary combustion)	Oxidizing	PFBA, PFPeA, PFHxA, PFHpA, PFOA, PFNA, PFDA, PFDoA, PFTeA, PFBS, FOSA, PFUnA, PFHxS, PFOS, NMeFOSAA, NEtFOSAA, 6:2 FTS
Major (2019)	PFOA, PFOS, PFHxS, PFNA, PFBS, and PFHpA	1,000°C	Oxidizing	PFBA, PFBS, PFPeA, PFHxA, PFHxS, PFHpA, PFOA, PFOS, PFNA, PFDA, PFUnA, PFDoA
Watanabe et al. (2018)	PFOA, PFHxA, PFOS (on GAC)	700°C	Reducing	Incomplete balance suggests volatile organofluoro compounds
Watanabe et al. (2016)	PFOA, PFHxA (on GAC) PFOS (on GAC)	800°C 900°C 1,000°C 700–1,000°C	Reducing	PFPeA, PFBA PFBA None None
García et al. (2007)	PTFE	750°C 850°C 950°C 1,050°C	Oxidizing	CF_4_, C_2_F_6_, benzoyl fluoride CF_4_, C_2_F_6_ CF_4_, C_2_F_6_, C_3_F_6_, benzenepentafluoro CF_4_, C_2_F_6_, benzenepentafluoro
Yamada et al. (2005)	Fluorotelomer‐based acrylic polymer (C_0.33_H_0.40_O_0.04_F_0.19_Cl_0.04_) Treated polyester fiber (2% F by weight)	600°C 1,000°C 725°C	Oxidizing	Flurobenzene, difluorobenzene, ·CF_3_, ·CF_2_CH=CH_2_ ·CF_3_, ·CF_2_CH=CH_2_ ·CF_3_
Krusic et al. (2005)	PFOA	307°C 355–385°C	Vacuum	Perfluoro‐1‐heptene 1‐H‐perfluoroheptane, perfluoro‐1‐heptene
Krusic and Roe (2004)	Ammonium PFOA	196–234°C	Vacuum	1‐H‐perfluoroheptane
Ellis et al. (2003)	PTFE	550°C	Oxidizing	fluoroacids
Taylor and Yamada (2003)	PFOS	600°C 900°C	Oxidizing	CF_4_ or C_2_F_6_ (postulated), 1,1‐difluroethene CF_4_ or C_2_F_6_ (postulated)
Ellis et al. (2001)	PTFE	500°C	Oxidizing	Tetrafluoroethene, hexafluoropropene, trifluoroacetate, cyclo‐octafluorobutane, CF_3_(CF_2_)_n_COOH, CF_3_O(CF_2_)_m_COOH, monofluoracetic acid, difluoroacetic acid
Conesa and Font (2001)	PTFE	700°C	Oxidizing	C_2_F_2_, C_2_F_4_, CF_4_, CHF_3_, CH_2_F_2_, C_2_F_6_, ·CFO, CH_2_F_2_, cyclo‐C_4_F_8_, C_3_F_3_
			Reducing	same
Simon and Kaminsky (1998)	PTFE	500–600°C	Reducing	Trifluroethylene, hexafluroproprene, cyclo‐octafluorobutane
Jun et al. (1995)	PTFE	510–600°C	Reducing	Tetrafluoroethylene, hexafluroproprene, cyclo‐octafluorobutane
Baker and Kasprzak (1993)	PTFE	400°C	Oxidizing Reducing	Tetrafluoroethylene, hexafluropropylene, trifluoroacetyl fluoride, fluoroform, perfluoroisobutylene hexafluropropylene, perfluoroisobutylene
Blake and Tomlinson (1971)	Fluoroacetic acid	295–382°C	Vacuum	Fluoroacetyl fluoride
Sheppard and Sharts (1969), Kissa (2001)	Perfluoroalkanoic acid	550°C	Not identified	Perfluoroalkenes, hydrogen fluoride
Perfluoroalkanoic potassium salt	165–200°C		Perfluoroalkenes
Scheel et al. (1968)	PTFE	550°C	Reducing	Carbonyl fluoride
Lewis and Naylor (1947)	PTFE	600–700°C	Reducing	Tetrafluoroethylene

^A^ Compound acronym definition can be found in the source reference.

These studies demonstrate that an array of by‐products is possible during thermal treatment of PFAS. Given the diverse chemical species potentially formed as by‐products, the currently available analytical methods based on a small number of targeted PFAS compounds are inadequate for following the fate of PFAS through thermal processes.

Little is currently known regarding the formation pathways for by‐products from PFAS combustion. Burgess et al. (1995) presented theoretical pathways for one‐ and two‐carbon fluorinated species. Even when limiting consideration to these two simplest PFAS, the possible degradation products and intermediates are extensive. The study considered the formation of longer‐chain compounds from simpler radicals suggesting reformation is possible. García et al. (2007) proposed PTFE would decompose to tetrafluroethene (C_2_F_4_) under reducing conditions and potentially reform as C_3_F_6_, which would then combust to CF_4_. No further discussion on reformation of PFAS or other compounds of concern from PFAS combustion was identified in available literature.

The formation of polychlorinated dibenzo‐p‐dioxins (PCDD) and polychlorinated dibenzofurans (PCDF) in incineration systems may provide a valuable analogy, keeping in mind that the bond energies reported in Table 2 indicate large differences between C–Cl and C–F bonds. The PCDD/PCDF compounds are typically not present in wastewater solids but are often detected in incinerator emissions. McKay (2002) provides an extensive review of PCDD/PCDF emissions from municipal solid waste incineration. Formation of PCDD/PCDF compounds was noted at approximately 400°C, which is not a typical temperature for SSI operations unless the facility includes a waste heat boiler. Two formation pathways likely for PCDD/PCDF formation include: (a) precursor compounds from incomplete combustion of chlorobenzenes and chlorophenols or (b) de novo pathway forming PCDD/PCDF from simpler compounds in the flue gas. Either pathway requires chlorine to halogenate the complex organic molecules. Furthermore, ash from wastewater incinerators has been shown to promote PCDD/PCDF formation if chlorine is present, or catalyze oxidation in its absence (Fullana et al., 2004). The complex chemistry in incineration systems may promote similar formations for PFAS‐related compounds and will require further study to determine pathways to measured end products.

## Prospective SSI PFAS Fate

Despite their reputation, PFAS compounds will combust during incineration. Early laboratory‐scale work by Taylor and Yamada (2003) showed that PFOS will combust in a nonturbulent laboratory‐scale reactor within the same temperature range that an SSI operates. Aleksandrov et al. (2019) conducted thermogravimetric analyses (TGA) of PTFE that exhibited complete destruction in <1 s at 800°C, similar to SSI temperature regimes. Khan et al. (2020) calculated a PFOS half‐life of 0.2 s at 726°C. MacGregor (2020) reported PFAS is being destroyed through an FBF SSI (830°C for 8 s) based on mass flows into and out of the incineration system though the stack emissions have yet to be characterized. At higher temperatures, more typical of hazardous waste incinerators, significant PFAS destruction has been reported for a variety of congeners (Focus Environmental Inc., 2020; Lemieux et al., 2007; Ministry of the Environment of Japan, 2013; NRC Alaska LLC, 2019; Taylor et al., 2014; Yamada et al., 2005). The existing published literature is too narrow to determine whether all PFAS combust under incineration conditions given analytical limitations. Further, the potential reduction in combustion efficiency of the studied PFAS at typical SSI temperatures has not been characterized. However, given the turbulent environments and long residence times of the FBF and MHF at least some destruction is expected to occur.

The type of PFAS may also determine combustion efficiency within an incinerator. In contrast to the PFAS mentioned in the previous paragraph, CF_4_ may require over 1,400°C for complete destruction (Tsang et al., 1998). In the case of the investigation by Loganathan et al. (2007), nonpolymeric PFAS (PFHxS, PFNA, PFOA, PFOS, PFOSA, PFDA, perfluoroundecanoic acid [PFUnDA], and perfluorododecanoic acid [PFDoDA]) would have likely dominated the incinerator feed, which resulted in some PIC levels in the ash. By comparison, both Lemieux et al. (2007) and Aleksandrov et al. (2019) studied systems deliberately incinerating polymeric forms of PFAS and did not observe any significant PIC formation. Likewise, Taylor et al. (2014) indicated the combustion of a fluorotelomer‐based polymer produced no PICs. While there is inadequate information available to verify a trend, polymeric PFAS may combust more efficiently than nonpolymer forms.

Formation of PICs during PFAS incineration at temperatures achieved in SSIs has not been well documented. In the SSI study by Loganathan et al. (2007), some ash samples reported higher levels of PFAS than that measured in the wastewater solids fed to the incinerator. If the volatile solids content of the wastewater solids fed to the incinerator was over 65% then despite the high concentrations in the ash the overall mass of each PFAS was reduced. Otherwise, this may be a result of sampling time and wastewater solid PFAS variability, transformation of precursor compounds to the measured PFAS, or sampling and analytical contamination. In studies of other combustion systems, contamination has been suggested as the source of quantifiable PICs in ash, air emissions, and scrubber water (Aleksandrov et al., 2019; Lemieux et al., 2007; NRC Alaska LLC, 2019). However, Sandblom (2014) and Solo‐Gabriele et al. (2020) reported ash PFAS levels from MSW incinerators operating with similar temperature ranges as SSIs. SSIs, especially the FBF, will promote more turbulence than a typical moving grate incinerator used in MSW incineration and may therefore increase the relative level of PFAS destruction. Lastly, the available work only concerns PFAS that can be quantified, leaving unaccounted the vast majority of PFAS. Overall, no incineration system is 100% efficient, but the level of PICs released by SSIs is likely low compared to other environmental PFAS contamination pathways and whatever reduction occurs results in an overall reduction in global PFAS environmental discharge.

Characterizing PICs will provide the necessary context for concern from both a public health and a greenhouse gas (GHG) perspective. For example, while CF_4_ has been identified as a potential PIC with a high GHG impact but no health concerns, potential emission rates from an SSI are low. If a large urban SSI facility processing 100 dry tons per day converted 50% of the PFAS load to CF_4_, the unchecked emissions would be equivalent to the emissions of one passenger vehicle. This estimate assumed the PFAS wastewater solids content based on New England Biosolids and Residuals Association (NEBRA) and New Hampshire Department of Environmental Services (NHDES) PFAS sampling data summarized by Rainey (2019). As environmental and legislative bodies have introduced calls to ban SSI operation due to concerns over the impact of PICs, understanding the makeup and levels emitted would provide necessary data for the developing regulatory landscape.

The PFAS content of wastewater solids and characteristics thereof must also be taken into consideration in context to other incineration operations. Incinerating PFAS compounds with hydrocarbon‐rich fuel sources may improve destruction efficiency. Narengerile et al. (2010) calculated the destruction of hydrofluorocarbons in thermal plasma with and without water. In the presence of water, which supplied hydrogen and oxygen radicals, fluorocarbon by‐products were eliminated. Watanabe et al. (2018) observed more than a 20% increase in recovered mineralized fluorine when adsorbed to GAC compared with thermally treating the PFAS reagents alone at 700°C in a nitrogen atmosphere. The chemistry with GAC or other hydrocarbon fuels may be responsible for the enhanced PFAS destruction. Wang et al. (2013) observed higher PFOS destruction rates when subjecting lime‐treated wastewater solids to 300°C compared with adding calcium hydroxide to PFOS alone (Wang et al., 2011). The authors speculated the enhanced thermal degradation could be due to catalyzing metals in the wastewater solids. Taylor and Yamada (2003) hypothesized that PFOS combustion with methane provides the required hydrogen atoms to scavenge fluorine radicals to prevent PFAS reformation. Yamada et al. (2005) observed combustion temperatures required for no PIC formation were lower with a PFAS‐impregnated textile compared with the PFAS alone. Because the level of PFAS in biosolids is several orders of magnitude less than that required for RCRA hazardous waste classification and lower than MSW (Sanborn Head, 2019), PFAS destruction through an SSI may outperform other incineration industries, especially given the characteristics of the wastewater solids.

For those SSIs using an adsorption technology for mercury removal (i.e., activated carbon or SPC), further PFAS removal is also expected. Activated carbon represents one of the main treatment technologies applied to aqueous streams (Horst et al., 2018). Activated carbon is widely used in incinerators, including SSIs, to remove organic pollutants such as PCDDs and PCDFs from flue gas (Niessen, 2002). The relatively new SPC technology marketed for mercury and sulfur dioxide removal may also remove PCDDs and PCDFs, which is just being explored (EnviroCare International & personal communication, 2020). The potential PFAS removal provided by these air pollution control technologies would further reduce environmental release from an SSI.

## Research Needs

The current scientific knowledge on the behavior of PFAS through thermal processes is limited and requires additional study to understand how to best address public concerns and best practices for disposing of contaminated material. A near‐term study on the fate of PFAS through SSIs, like the study being finalized by MacGregor (2020) but looking at both MHF and FBF technologies is crucial and recommended. Sampling and analytical techniques that are available for identifying a broad spectrum of PFAS will be useful in developing a materials balance for such a study (Winchell et al., in review). While these initial full‐scale studies will provide the gross fate of PFAS through SSIs and assist regulators and policy makers to respond to public pressure, additional research will be required to better understand the mechanisms behind the observed SSI performance and subsequently develop implementable solutions.

One of the more basic areas of research needed is identifying the diversity of PFAS present in wastewater solids. As noted in Table 2, theoretical calculations (Burgess et al., 1995; Tsang et al., 1998) and laboratory data (Taylor & Yamada, 2003; Yamada et al., 2005) both illustrate the impacts of PFAS chemical structure on thermal degradation. Simply stated, thermal conditions for destruction will in part depend on the specific PFAS present. As analytical techniques develop, improved characterization of the PFAS in wastewater solids will provide the industry with key compounds to target in development and operation of treatment methods.

Additional research must also focus on the nature of thermal degradation of PFAS under SSI operating conditions. Existing research has largely focused on operating conditions in hazardous waste incinerators, which differ from SSIs. Table 1 summarizes the different operating conditions between several types of incinerators. Although SSIs operate at lower temperatures than hazardous waste facilities, the SSI often use longer residence times and higher turbulence critical to PFAS destruction. Laboratory and pilot‐scale research focused on MHF and FBF technologies, under controlled and representative conditions, would provide immensely valuable information on PFAS destruction efficiency. In part, these scaled down studies can evaluate different operating conditions for optimizing PFAS destruction without creating issues with other pollutant emissions or treatment goals.

Additional research should also focus on the complex combustion chemistry resulting from use of wastewater solids as a fuel source. Several studies have indicated using hydrocarbon fuel sources increases PFAS destruction efficiency (Taylor & Yamada, 2003; Yamada et al., 2005). Others observed positive impacts from the addition of calcium (Wang et al., 2013). Further research may identify catalysts to enhance PFAS destruction and lead to engineered solutions.

Emerging thermal treatment technologies, such as hydrothermal liquefaction or pyrolysis, operate under different conditions compared with existing SSIs. Given these technologies are under active development, their performance regarding PFAS destruction is also unknown. These technologies require separate research directives but can also be complementary to SSI research. Based on the emerging technologies discussed in this review, one key area differentiated from SSIs will be PFAS thermal degradation in the absence of or in substoichiometric oxygen conditions.

## Conclusions

Thermal treatment of PFAS through an SSI represents a potential wastewater solids process for destroying PFAS; however, significant questions remain regarding both the destruction efficiency and potential formation of undesirable by‐products. While nearly complete PFAS decomposition has been demonstrated at temperatures representative of SSI operation, by‐products have also been observed. Temperature is only one of the three primary parameters when assessing destruction capacity in combustion systems, the other two being residence time and turbulence. A well‐functioning SSI will submit PFAS to greater residence times and mixing (or turbulence) than the laboratory‐scale research performed to date, further promoting PFAS destruction. If PFAS parent compounds are recalcitrant or PICs are formed, they will be subjected to air pollution control equipment, which will likely capture an additional fraction of PFAS compounds.

Consequently, a critical near‐term need exists to evaluate the fate of PFAS through full‐scale SSIs to understand the fate of the PFAS in the wastewater solids and identify PICs in stack emissions and air pollution control residual streams. Initial testing should focus on sites representative of SSI industry operating conditions and configurations. Subsequent full‐scale testing must consider operational changes or various air pollution control technologies to minimize PFAS emissions. Furthermore, an extensive database on PFAS content in wastewater solids at incineration facilities would inform the utilities of their PFAS loadings. Results should be compared among studied emissions to ascertain site‐specific risks. Any of these research objectives must incorporate emerging analytical techniques to characterize the PFAS to the fullest extent, while using sampling techniques capable of collecting polar and nonpolar, as well as volatile, nonvolatile, and semi‐volatile PICs. A comprehensive review of the sampling and analytical techniques available for utilizing these emerging techniques has been prepared separately (Winchell et al., in review).

## Conflicts of Interest

In submitting this manuscript, the authors do not have any conflicts of interest or other considerations that would limit publication of the manuscript. Martha J.M. Wells is a chemical consultant to Brown and Caldwell and served in that role while assisting with preparation of this manuscript.

## Data Availability

Data sharing is not applicable to this article as no datasets were generated or analyzed during the current study.
